# Longitudinal Analysis of Neighborhood Food Environment and Diabetes Risk in the Veterans Administration Diabetes Risk Cohort

**DOI:** 10.1001/jamanetworkopen.2021.30789

**Published:** 2021-10-29

**Authors:** Rania Kanchi, Priscilla Lopez, Pasquale E. Rummo, David C. Lee, Samrachana Adhikari, Mark D. Schwartz, Sanja Avramovic, Karen R. Siegel, Deborah B. Rolka, Giuseppina Imperatore, Brian Elbel, Lorna E. Thorpe

**Affiliations:** 1 Department of Population Health, NYU Langone Health, New York, New York; 2 Department of Emergency Medicine, NYU Langone Health, New York, New York; 3 VA New York Harbor Healthcare System, New York, New York; 4 Department of Health Administration and Policy, George Mason University, Fairfax, Virginia; 5 Division of Diabetes Translation, Centers for Disease Control and Prevention, Atlanta, Georgia; 6 NYU Wagner Graduate School of Public Service, New York, New York

## Abstract

**Importance:**

Diabetes causes substantial morbidity and mortality among adults in the US, yet its incidence varies across the country, suggesting that neighborhood factors are associated with geographical disparities in diabetes.

**Objective:**

To examine the association between neighborhood food environment and risk of incident type 2 diabetes across different community types (high-density urban, low-density urban, suburban, and rural).

**Design, Setting, and Participants:**

This is a national cohort study of 4 100 650 US veterans without type 2 diabetes. Participants entered the cohort between 2008 and 2016 and were followed up through 2018. The median (IQR) duration of follow-up was 5.5 (2.6-9.8) person-years. Data were obtained from Veterans Affairs electronic health records. Incident type 2 diabetes was defined as 2 encounters with type 2 diabetes *International Classification of Diseases, Ninth Revision or Tenth Revision* codes, a prescription for diabetes medication other than metformin or acarbose alone, or 1 encounter with type 2 diabetes *International Classification of Diseases Ninth Revision or Tenth Revision* codes and 2 instances of elevated hemoglobin A_1c_ (≥6.5%). Data analysis was performed from October 2020 to March 2021.

**Exposures:**

Five-year mean counts of fast-food restaurants and supermarkets relative to other food outlets at baseline were used to generate neighborhood food environment measures. The association between food environment and time to incident diabetes was examined using piecewise exponential models with 2-year interval of person-time and county-level random effects stratifying by community types.

**Results:**

The mean (SD) age of cohort participants was 59.4 (17.2) years. Most of the participants were non-Hispanic White (2 783 756 participants [76.3%]) and male (3 779 555 participants [92.2%]). The relative density of fast-food restaurants was positively associated with a modestly increased risk of type 2 diabetes in all community types. The adjusted hazard ratio (aHR) was 1.01 (95% CI, 1.00-1.02) in high-density urban communities, 1.01 (95% CI, 1.01-1.01) in low-density urban communities, 1.02 (95% CI, 1.01-1.03) in suburban communities, and 1.01 (95% CI, 1.01-1.02) in rural communities. The relative density of supermarkets was associated with lower type 2 diabetes risk only in suburban (aHR, 0.97; 95% CI, 0.96-0.99) and rural (aHR, 0.99; 95% CI, 0.98-0.99) communities.

**Conclusions and Relevance:**

These findings suggest that neighborhood food environment measures are associated with type 2 diabetes among US veterans in multiple community types and that food environments are potential avenues for action to address the burden of diabetes. Tailored interventions targeting the availability of supermarkets may be associated with reduced diabetes risk, particularly in suburban and rural communities, whereas restrictions on fast-food restaurants may help in all community types.

## Key Points

**Question:**

Is there an association between the presence of fast-food restaurants and availability of supermarkets in neighborhoods and the risk of developing type 2 diabetes?

**Findings:**

In this longitudinal cohort study of 4 100 650 veterans, the relative availability of fast-food restaurants compared with all restaurants within neighborhoods was associated with an increased risk of diabetes across a spectrum of rural to high-density urban settings, whereas the availability of supermarkets had an inverse association with incident diabetes in suburban and rural communities only.

**Meaning:**

These findings suggest that policies to shift the mix of fast-food restaurant and supermarket distribution in neighborhoods may be associated with reduced diabetes risk.

## Teaser

This cohort study examines the association between neighborhood food environment and risk of type 2 diabetes across different community types in the Veterans Administration Diabetes Risk (VADR) cohort.

## Introduction

Diabetes is a major cause of morbidity and mortality in the US.^1,2^ In 2018, the Centers for Disease Control and Prevention estimated that 34.2 million adults aged 18 years and older in the US (13% of the population) had diabetes.^1^ Although the risk of diabetes has increased in all parts of the country since the late 1990s, there are substantial geographical disparities in diabetes prevalence and incidence.^1,3,4^ County-level analyses have highlighted age-adjusted prevalence of diabetes ranging from 1.5% (mostly counties in the West) to as high as 33.0% in others (mostly counties in the Southeast).^1^ Substantial geographical disparities have also been observed between neighboring counties with similar demographic profiles, suggesting a heterogeneous impact of neighborhood-level factors on diabetes.^5,6,7,8^

A growing body of literature has examined the role of the food environment on the risk of diabetes.^9,10,11,12,13^ Longitudinal studies have found that better neighborhood resources, such as suitability for physical activity and availability of healthy food, were associated with reduced diabetes risk, whereas higher density of food stores selling less healthy foods was associated with increased diabetes risk, likely related to behavioral changes due to changes in access to neighborhood resources. However, these studies had limited geography to a handful of urban environments, thus limiting generalizability and prohibiting exploration of how such associations vary across other environments (ie, urban, suburban, or rural settings). The small size of this research body and methodological variability in (1) data sources and definitions of food environment and (2) methods used to operationalize how food environments are measured by urbanicity, as well as insufficient geographical scope in published studies to date, has restricted our ability to understand and characterize the association between the food environment and diabetes across the US. To our knowledge, no study to date has examined associations between neighborhood food environment measures and diabetes incidence using objectively measured food establishment data at the US Census tract level nationwide while stratifying by urbanicity.

The Diabetes Location, Environmental Attributes, and Disparities (Diabetes LEAD) network is a Centers for Disease Control and Prevention–funded collaboration between multiple academic institutions aiming to study the role of community level factors on diabetes incidence.^14^ We explored the association of neighborhood food environments, specifically the presence of fast-food establishments and supermarkets, on type 2 diabetes incidence among a cohort of US veterans using the Veteran’s Affairs (VA) electronic health record (EHR).

## Methods

### Individual-Level Data

This cohort study was approved by New York University and the VA institutional review boards, which waived the need for informed consent given the retrospective nature of the study and deidentified data, in accordance with 45 CFR §46. This study followed the Strengthening the Reporting of Observational Studies in Epidemiology (STROBE) reporting guidelines.

Data used for this study are from the US Veterans Administration Diabetes Risk (VADR) cohort, a cohort of veterans without type 2 diabetes constructed by the New York University Grossman School of Medicine and George Mason University through the VA national EHR.^15^ The VADR is a national cohort of US veterans enrolled in the VA for primary care. Veterans were passively enrolled into VADR if they did not have type 2 diabetes as of January 1, 2008, and had at least 2 primary care visits at least 30 days apart within any 5-year period since January 1, 2008, through December 31, 2016. Patients were considered to have type 2 diabetes and were excluded if they met the following criteria before entering the cohort: 2 encounters with *International Classification of Diseases, Ninth Revision* (*ICD-9*) or *International Statistical Classification of Diseases and Related Health Problems, Tenth Revision* (*ICD-10*) codes for type 2 diabetes, a prescription for type 2 diabetes medication other than metformin or acarbose alone, or 1 inpatient or outpatient encounter with *ICD-9* or *ICD-10* type 2 diabetes codes and 2 instances of elevated hemoglobin A_1C_ (≥6.5% [to convert to proportion of total hemoglobin, multiply by 0.01]). The cohort enrolled 6 082 018 veterans and observed them through December 31, 2018; the median (IQR) duration of follow-up was 5.5 (2.6-9.8) person-years. Person-time was calculated as the interval between cohort entry date and incident diabetes or censor date. Veterans were censored if they died or did not have encounters with the Veterans Health Administration for 2 years (lost to follow-up).

Addresses were geocoded using ArcGIS by Esri.^16^ Post office box addresses and addresses with missing information (1 032 944 addresses) and individuals whose addresses were not located in the continental US (63 443 addresses) were excluded. Patients with first documented address occurring more than 2 years after cohort entry date and with an inconsistent history of clinic visits were excluded because of unclear information to consider these addresses as baseline addresses (884 981 addresses). Valid baseline addresses’ geolocation identifications were linked to neighborhood characteristics obtained from the Retail Environment and Cardiovascular Disease study^17,18^ and the American Community Survey.^19^ More information about the final cohort has been published elsewhere.^15^

### Definitions

Age at baseline was calculated by subtracting the date of birth from cohort entry date. The following sex groups were reported: male and female. Race and ethnicity were categorized into Hispanic, non-Hispanic Asian, non-Hispanic American Indian or Alaska Native, non-Hispanic Black, non-Hispanic Native Hawaiian or other Pacific Islander, and non-Hispanic White. Race and ethnicity were derived from the VA EHR and were assessed in this study because of the persistent associations of race and ethnicity with health outcomes. Patients in the VA EHR are assigned to different priority groups on the basis of their military service history, disability, income, and eligibility for Medicaid or other VA benefits.^20^ These priority groups were used to create a low-income or disability flag that was used as a proxy for socioeconomic status. The low-income or disability flag was categorized hierarchically as with a disability, low-income but without a disability, and none of the above.

### Neighborhood-Level Data

All baseline neighborhood-level attributes were defined at cohort entry date. Relative food environment measures were identified as the primary exposures and included (1) the percentage of total food-serving establishments that were fast-food establishments, calculated by taking the 5-year mean number of fast-food restaurants in US Census tracts relative to all restaurants, and (2) the proportion of total retail food outlets that were supermarkets, also calculated by taking the 5-year mean number of supermarkets in US census tracts with assigned buffers relative to other food stores. Information on how these measures were created have been published elsewhere.^18,21^ A sensitivity analysis was done using absolute food environment measures, defined as 5-year mean fast-food restaurants and supermarkets density (count per square kilometer) to test the robustness of our models. All US Census tracts were categorized by the Diabetes LEAD network into 1 of 4 community types using a modified measure of the 2010 Rural-Urban Commuting Area: high-density urban (HDU), low-density urban (LDU), suburban or small town (suburban), and rural.^17^ Neighborhood food characteristics were assigned according to the availability of each type of food outlet within buffers created around US Census tracts. Buffer sizes were determined by the Diabetes LEAD network group through a consensus of experts as follows: 1-mile walking buffer for HDU communities, 2-mile driving buffer for LDU communities, 6-mile driving buffer for suburban communities, and 10-mile driving buffer for rural communities.^22^ Assigning food environment variables to addresses using each community type’s information with different buffers helped standardize these variables and made it feasible to compare findings between different community types.

Other neighborhood-level covariates were generated by the Diabetes LEAD network and included in the models. Neighborhood social and economic environment (NSEE) was created as a community type–stratified *z *score sum of the following census-derived measures from the American Community Survey: percentage of adults with less than a high school education, percentage of unemployed adults, percentage of households with annual income less than $30 000, percentage of households in poverty, percentage of households receiving public assistance, and percentage of households with no cars. NSEE was categorized into quartiles, with the first quartile being least disadvantaged neighborhoods and the fourth quartile being most disadvantaged neighborhoods. To account for neighborhood development compact level, the network created a land use environment variable based on mean block length, mean block size, intersection density, street connectivity, establishment density, percentage of developed land, and household density. More information about these neighborhood measures and the rationale for buffer sizes used has been published elsewhere.^17^ Finally, to adjust for neighborhood demographic confounders that could influence the built environment and health outcomes, 2 US Census tract demographic measures from the American Community Survey—percentages of Hispanic and non-Hispanic Black residents—were included in the models as continuous variables.

### Statistical Analysis

Neighborhood-level covariates and type 2 diabetes incidence were described in the full sample and also stratified by community type. To estimate the associations of the food environment with type 2 diabetes risk, adjusted hazard ratios (HRs) and 95% CIs were calculated using piecewise exponential (PWE) models with 2-year intervals of person-time and county-level random effects.^23^ One-year intervals were used as a sensitivity analysis, and the models yielded similar results (data not shown). PWE models assume that the hazard of the outcome is constant across intervals, which means that follow-up times follow an exponential distribution within each interval. Two separate models were fitted: the first with the 2 relative food measures and the second with the 2 absolute density food measures. The models also adjusted for individual (age [continuous], sex, race and ethnicity, and disability or low-income flag) and neighborhood-level (NSEE quartiles, land use environment, percentage non-Hispanic Black residents, percentage Hispanic residents, and food environment) covariates. Even though weight is highly correlated with diabetes, we decided not to include it in our models for being on the causal pathway between food and type 2 diabetes. Only individuals with available data on all covariates were included in the PWE models. A sensitivity analysis was done stratifying by region (Northeast, South, Midwest, and West). Models were fitted in the full sample and stratified by community types. Two-tailed *t* tests were used to assess significance, which was set at a threshold of *P* < .05. Statistical analyses were done using SAS statistical software version 9.4 (SAS Institute). Data analysis was performed from October 2020 to March 2021.

## Results

A total of 4 100 650 individuals were included in the analysis. The mean (SD) age at enrollment was 59.4 (17.2) years; veterans living in rural communities were slightly older than those living in urban communities (mean [SD] age, 60.2 [16.6] years vs 58.0 [17.4] years for HUD communities and 59.3 [17.6] years for LUD communities) (Table 1). Most of the cohort was male (3 779 555 participants [92.2%]) and the majority were non-Hispanic White (2 783 756 participants [76.3%]), including 53.3% (227 518 participants) in HDU communities, 71.3% (954 118 participants) in LDU communities, 80.7% (660 917 participants) in suburban communities, and 88.4% (941 203 participants) in rural communities. More than 70% of the cohort in all community types had either disability (1 403 858 participants [34.8%]) or low income (1 527 258 participants [37.9%]).

**Table 1.** Selected Demographic Characteristics of the Veterans Administration Diabetes Risk Cohort

Characteristic	Participants, No. (%)				
	All community types (N = 4 100 650)	High-density urban (n = 478 668)	Low-density urban (n = 1 509 042)	Suburban or small town (n = 919 281)	Rural (n = 1 193 659)
Individual-level variables					
Age, mean (SD), y	59.4 (17.2)	58.0 (17.4)	59.3 (17.6)	59.2 (17.4)	60.2 (16.6)
Age categories, y					
19-39	648 259 (15.8)	83 954 (17.5)	246 778 (16.4)	149 709 (16.3)	167 818 (14.1)
40-59	1 193 915 (29.1)	157 622 (32.9)	448 217 (29.7)	262 972 (28.6)	325 104 (27.2)
60-79	1 702 191 (41.5)	174 916 (36.5)	592 489 (39.3)	383 002 (41.7)	551 784 (46.2)
≥80	556 227 (13.6)	62 167 (13.0)	221 531 (14.7)	123 585 (13.4)	148 944 (12.5)
Sex					
Male	3 779 555 (92.2)	437 088 (91.3)	1 379 862 (91.4)	846 901 (92.1)	1 115 704 (93.5)
Female	321 013 (7.8)	41 564 (8.7)	129 145 (8.6)	72 363 (7.9)	77 941 (6.5)
Race and ethnicity					
Hispanic	189 177 (5.2)	44 340 (10.4)	86 580 (6.5)	35 380 (4.3)	22 877 (2.2)
Non-Hispanic					
American Indian or Alaska Native	28 327 (0.8)	2822 (0.7)	9188 (0.7)	6141 (0.8)	10 326 (1.0)
Asian	34 838 (1.0)	10 464 (2.5)	15 751 (1.2)	6141 (0.8)	2482 (0.2)
Black	584 655 (16.0)	137 039 (32.1)	261 417 (19.5)	104 592 (12.8)	81 607 (7.7)
Native Hawaiian or other Pacific Islander	28 477 (0.8)	4465 (1.1)	11 748 (0.9)	6153 (0.8)	5961 (0.6)
White	2 783 756 (76.3)	227 518 (53.3)	954 118 (71.3)	660 917 (80.7)	941 203 (88.4)
Income or disability					
Disability	1 403 858 (34.8)	143 787 (30.7)	529 283 (35.7)	335 036 (37.0)	395 752 (33.7)
Low income	1 527 258 (37.9)	225 099 (48.0)	560 775 (37.8)	308 897 (34.1)	432 487 (36.8)
None of the above	1 100 899 (27.3)	99 760 (21.3)	391 821 (26.4)	261 325 (28.9)	347 993 (29.6)
Neighborhood-level variables					
Relative fast-food restaurants, mean (SD), %	0.30 (0.13)	0.26 (0.14)	0.31 (0.13)	0.32 (0.10)	0.29 (0.15)
Relative supermarkets, mean (SD), %	0.11 (0.07)	0.09 (0.08)	0.10 (0.08)	0.11 (0.05)	0.12 (0.08)
NSEE continuous, mean (SD)	16.1 (9.6)	23.6 (13.1)	13.2 (8.6)	13.3 (8.1)	18.9 (7.5)
NSEE quartiles					
First (most advantaged)	947 002 (23.1)	126 567 (26.5)	320 370 (21.2)	209 164 (22.8)	290 901 (24.4)
Second	1 130 576 (27.6)	135 038 (28.2)	411 960 (27.3)	262 205 (28.5)	321 373 (26.9)
Third	1 132 148 (27.6)	115 778 (24.2)	430 590 (28.6)	263 587 (28.7)	322 193 (27.0)
Fourth (least advantaged)	889 432 (21.7)	100 868 (21.1)	345 444 (22.9)	184 071 (20.0)	259 049 (21.7)
Land use environment, mean (SD)	0.01 (0.91)	−0.06 (0.83)	0.01 (0.92)	−0.05 (0.93)	0.09 (0.92)
Hispanic residents, mean (SD), %	10.2 (16.0)	20.2 (23.1)	12.3 (16.7)	8.2 (13.4)	4.9 (9.7)
Non-Hispanic Black residents, mean (SD), %	12.7 (21.3)	26.1 (32.5)	15.1 (22.7)	9.3 (15.3)	6.9 (13.5)

Abbreviation: NSEE, neighborhood social and economic environment.

During follow-up, 13.2% of the cohort (539 369 participants) met the criteria for type 2 diabetes incidence. Cumulative incidence was highest among those aged 60 to 79 years (288 836 participants [17.0%]), followed by those aged 40 to 59 years (178 302 participants [14.9%]). In univariate analysis, 13.6% of men (512 920 participants) developed type 2 diabetes compared with 8.2% of women (26 439 participants). Non-Hispanic Black adults had the highest incidence of type 2 diabetes (99 013 participants [16.9%]) compared with other racial and ethnic groups (4250 non-Hispanic Native Hawaiian and other Pacific Islander participants [15.0%], 4046 non-Hispanic American Indian and Alaska Native participants [14.2%], 359 649 non-Hispanic White participants [12.9%], and 4473 non-Hispanic Asian and 24 236 Hispanic participants [12.8% each]). Adults with disability and those with low income but no disability had higher incidence (192 341 participants [13.7%] and 214 927 participants [14.1%], respectively) than those with neither disability nor low income (127 074 participants [11.5%]). The proportion of adults with type 2 diabetes increased as the NSEE quartiles moved from the most advantaged to the least advantaged (112 131 participants [11.8%] vs 131 638 participants [14.8%]) (Table 2). When stratifying by community types, the proportion of adults with type 2 diabetes was highest among those living in HDU communities (14.3%; 95% CI, 14.2%-14.4%) followed by those living in LDU (13.1%; 95% CI, 13.0%-13.1%) and rural (13.2%; 95% CI, 13.2%-13.3%) communities and was lowest among those living in suburban communities (12.6%; 95% CI, 12.5%-12.6%). The patterns observed by individual characteristics were also observed by community type stratum.

**Table 2.** Incidence of Type 2 Diabetes by Demographic Characteristics, Overall and by Community Type

Variable	All community types		High-density urban		Low-density urban		Suburban or small town		Rural	
	Participants, No.	Incidence, % (95% CI)	Participants, No.	Incidence, % (95% CI)	Participants, No.	Incidence, % (95% CI)	Participants, No.	Incidence, % (95% CI)	Participants, No.	Incidence, % (95% CI)
Individual-level	539 369	13.2 (13.1-13.2)	68 286	14.3 (14.2-14.4)	197 583	13.1 (13.0-13.1)	11 603	12.6 (12.5-12.6)	157 897	13.2 (13.2-13.3)
Age categories, y										
19-39	21 131	3.3 (3.2-3.3)	2863	3.4 (3.3-3.5)	8441	3.4 (3.3-3.5)	4671	3.1 (3.0-3.2)	5156	3.1 (3.0-3.2)
40-59	178 302	14.9 (14.9-15.0)	26 289	16.7 (16.5-16.9)	67 774	15.1 (15.0-15.2)	37 076	14.1 (14.0-14.2)	47 163	14.5 (14.4-14.6)
60-79	288 836	17.0 (16.9-17.0)	32 555	18.6 (18.4-18.8)	101 285	17.1 (17.0-17.2)	62 840	16.4 (16.3-16.5)	92 156	16.7 (16.6-16.8)
≥80	51 096	9.2 (9.1-9.3)	6577	10.6 (10.3-10.8)	20 082	9.1 (8.9-9.2)	11 015	8.9 (8.8-9.1)	13 422	9.0 (8.9-9.2)
Sex										
Male	512 920	13.6 (13.5-13.6)	64 485	14.8 (14.6-14.9)	186 691	13.5 (13.5-13.6)	109 884	13.0 (12.9-13.0)	151 860	13.6 (13.5-13.7)
Female	26 439	8.2 (8.1-8.3)	3797	9.1 (8.9-9.4)	10 890	8.4 (8.3-8.6)	5717	7.9 (7.7-8.1)	6035	7.7 (7.6-7.9)
Race and ethnicity										
Hispanic	24 236	12.8 (12.7-13.0)	5684	12.8 (12.5-13.1)	11 099	12.8 (12.6-13.0)	4332	12.2 (11.9-12.6)	3121	13.6 (13.2-14.1)
Non-Hispanic										
American Indian or Alaska Native	4046	14.2 (13.8-14.6)	407	14.4 (13.1-15.7)	1275	13.9 (13.2-14.6)	809	13.2 (12.3-14.0)	1555	15.1 (14.4-15.7)
Asian	4473	12.8 (12.5-13.2)	1396	13.3 (12.7-14.0)	2036	12.9 (12.4-13.5)	771	12.6 (11.7-13.4)	270	10.9 (9.7-12.1)
Black	99 013	16.9 (16.8-17.0)	24 457	17.8 (17.6-18.0)	44 163	16.9 (16.8-17.0)	16 712	16.0 (15.8-16.2)	13 681	16.8 (16.5-17.0)
Native Hawaiian or other Pacific Islander	4250	15.0 (14.6-15.4)	716	16.0 (15.0-17.1)	1738	14.8 (14.2-15.4)	868	14.1 (13.2-15.0)	928	15.6 (14.6-16.5)
White	359 649	12.9 (12.9-13.0)	30 388	13.4 (13.2-13.5)	121 161	12.7 (12.6-12.8)	82,752	12.5 (12.4-12.6)	125 348	13.3 (13.2-13.4)
Income and disability										
Disability	192 341	13.7 (13.6-13.8)	21 635	15.0 (14.9-15.2)	72 770	13.7 (13.7-13.8)	43 542	13.0 (12.9-13.1)	54 394	13.7 (13.6-13.9)
Low income	214 927	14.1 (14.0-14.1)	34 199	15.2 (15.0-15.3)	78 847	14.1 (14.0-14.2)	41 602	13.5 (13.3-13.6)	60 279	13.9 (13.8-14.0)
None of the above	127 074	11.5 (11.5-11.6)	11 729	11.8 (11.6-12.0)	44 083	11.3 (11.2-11.3)	29 384	11.2 (11.1-11.4)	41 878	12.0 (11.9-12.1)
Neighborhood level										
NSEE quartiles										
First (most advantaged)	112 131	11.8 (11.8-11.9)	15 792	12.5 (12.3-12.7)	36 916	11.5 (11.4-11.6)	23 875	11.4 (11.3-11.6)	35 548	12.2 (12.1-12.3)
Second	143 403	12.7 (12.6-12.7)	18 662	13.8 (13.6-14.0)	51 285	12.4 (12.3-12.5)	32 080	12.2 (12.1-12.4)	41 376	12.9 (12.8-13.0)
Third	152 035	13.4 (13.4-13.5)	17 372	15.0 (14.8-15.2)	57 129	13.3 (13.2-13.4)	33 849	12.8 (12.7-13.0)	43 685	13.6 (13.4-13.7)
Fourth (least advantaged)	131 638	14.8 (14.7-14.9)	16 423	16.3 (16.1-16.5)	52 166	15.1 (15-15.2)	25 778	14.0 (13.8-14.2)	37 271	14.4 (14.3-14.5)

Abbreviation: NSEE, neighborhood social and economic environment.

Approximately one-fourth of veterans lived in the most advantaged quartile of neighborhoods in all community types. Nearly one-third (mean [SD], 30% [0.13%]) of food-serving establishments were fast-food restaurants. The mean (SD) proportion of fast-food restaurants compared with other food outlets was 26% (14%) in HDU communities, 31% (13%) in LDU communities, 32% (10%) in suburban communities, and 29% (15%) in rural communities. The proportion of supermarkets compared with other retail food outlets was lower than fast-food restaurants and was approximately equal across community types (approximately 10%).

PWE model results showed that the proportion of baseline fast-food restaurants compared with all restaurants was associated with an increased risk of type 2 diabetes incidence in all 4 community types. A 10% increase in the number of fast-food restaurants compared with all restaurants was associated with a 1% increase in type 2 diabetes risk in HDU (aHR, 1.01; 95% CI, 1.00-1.02), LDU (aHR, 1.01; 95% CI, 1.01-1.01), and rural (aHR, 1.01; 95% CI, 1.01-1.02) communites, and a 2% increase in risk of type 2 diabetes (aHR, 1.02; 95% CI, 1.01-1.03) in suburban communities (Figure). In contrast, a 10% increase in supermarket density compared with other food stores was associated with lower risk of type 2 diabetes in suburban (aHR, 0.97; 95% CI, 0.96-0.99) and rural (aHR, 0.99; 95% CI, 0.98-0.99) communities, but the association was not significant in both types of urban communities (Figure).

**Figure.**
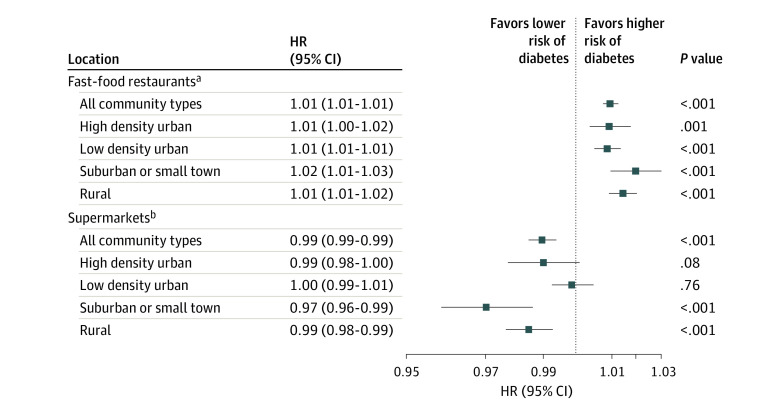
Piecewise Exponential Models Testing the Association of the Proportion of Fast-Food Restaurants Relative to All Restaurants and Supermarkets Relative to All Food Stores in Neighborhood With the Risk of Developing Type 2 Diabetes Among US Veterans, 2008-2018 Only 3 601 526 individuals with available data on all the variables in the models are included. HR indicates hazard ratio. ^a^Model was adjusted for age, sex, race and ethnicity, low-income or disability flag, neighborhood social and economic environment, land use environment, percentage of Hispanic and Black residents in the neighborhood, and 5-year mean supermarket count relative to all food stores in network buffers. ^b^Model was adjusted for age, sex, race and ethnicity, low-income or disability flag, neighborhood social and economic environment, land use environment, percentage of Hispanic and Black residents in the neighborhood, and 5-year mean fast food restaurant count relative to all restaurants in network buffers.

When absolute measures of neighborhood fast-food restaurant and supermarket density were used instead of relative food environment measures, findings were slightly different for some community types. Fast-food restaurant density was no longer associated with type 2 diabetes risk in HDU communities. Increased supermarket density was associated with a lower risk of type 2 diabetes among individuals living in HDU communities (aHR, 0.996; 95% CI, 0.995-0.997) in addition to suburban (aHR, 0.974; 95% CI, 0.961-0.987) and rural (aHR, 0.912; 95% CI, 0.880-0.946) communities (eFigure 1, eFigure 2, and eAppendix in the Supplement). Sensitivity analyses stratifying PWE models by region in addition to community types yielded similar results, suggesting that there was no need to stratify by region (data not shown).

## Discussion

This cohort study of US veterans is the first, to our knowledge, to prospectively examine the association between neighborhood food environment and type 2 diabetes risk nationally and by community type, using exposure measures tailored to community type. The availability of fast-food restaurants relative to all restaurants was associated with a higher risk of type 2 diabetes in all community types, whereas supermarkets were associated with a lower type 2 diabetes risk in suburban and rural communities. Our models did not find a significant association between supermarket availability and type 2 diabetes incidence in urban communities.

To date, only a limited number of studies^24,25^ have examined the association between neighborhood food environment and type 2 diabetes incidence using longitudinal data. Our study found that the association of food environment with type 2 diabetes incidence varied by level of urbanicity but did not vary further by region. Other studies^24,25^ focused only on urban communities reported an association between type 2 diabetes incidence and food environment that was different from urban communities strata findings from our cohort. One study, using data from the Multi-Ethnic Study of Atherosclerosis, found that better neighborhood resources was associated with a reduced risk of type 2 diabetes incidence (HR, 0.62; 95% CI, 0.43-0.88),^24^ but in this case access to neighborhood resources combined both physical activity and healthy food establishments. Another study, using data from the Jackson Heart Study, found that higher density of unfavorable food stores was associated with a higher risk of type 2 diabetes incidence (HR, 1.34; 95% CI, 1.12-1.61).^25^ This association was larger than the association we found in urban communities. However, the data used were geographically focused in and around a single urban area.^25^ Another study^26^ using a cross-sectional analysis and self-reported food environment found no significant association of food environment with insulin resistance and type 2 diabetes.

Unlike other community types, our findings suggest that the relative availability of supermarkets in urban communities was not associated with type 2 diabetes. This could be explained by access to both public transportation and cars, specifically in LDU communities, which could increase the ability to access supermarkets, regardless of availability within the residential neighborhoods. Thus, interventions targeting the placement or zoning of supermarkets may be more appropriate in suburban and rural communities.

In our study, the association between the relative availability of fast-food restaurants and type 2 diabetes incidence was similar in all community types. Results from our sensitivity analysis indicated that the association between the absolute availability of fast-food restaurants and type 2 diabetes incidence was larger in suburban and rural communities compared with LDU communities and was null in HDU communities. Given the high population density in HDU communities, the absolute count of food outlets per kilometer may mirror population density, rather than quality of food environment. In addition, these urban centers have higher socioeconomic status than other areas in HDU communities. Taken together, our findings suggest that policies specific to fast-food restaurants, such as policies restricting the siting of fast-food restaurants and healthy beverage default laws,^27,28^ may be effective in reducing type 2 diabetes risk in all community types. In urban areas where population and retail density are growing, it will be even more important to focus on these policies.

### Strengths and Limitations

Strengths of this study include examining the association between type 2 diabetes and neighborhood food environment in a large, national, longitudinal cohort using robust statistical methods. PWE models allowed us to test the longitudinal association of food environment with the risk of incident type 2 diabetes, accounting for the multilevel structure of the data and stratifying by different community types. Neighborhood characteristics were assigned using walking and driving buffers around individuals’ addresses, the buffer sizes of which were designed to be congruent with community types.

This study has several limitations. The analysis was observational, and the exposure was not randomly assigned to participants. Given that the study used EHR data, we were unable to capture residual lifestyle confounders, such as diet, physical activity, and comorbidities. Follow-up frequency was not constant across all cohort participants. The study may also not be generalizable to nonveteran populations; US veterans have substantially greater financial and health burdens than the civilian population and are at an increased risk of disability, obesity, and other chronic conditions.^29,30^ Although most of the cohort was composed of non-Hispanic White men, it did also include a sizable number of women and participants of other races and ethnicities, which were included in our models. We were unable to account for participants’ individual household income; however, a low-income or disability flag was used as a proxy for socioeconomic status, and we further adjusted for neighborhood-level socioeconomic factors. Neighborhood characteristics were assigned using patients’ baseline address regardless of the possibility of moving. However, it was previously found that even if people move, they tend to live in neighborhoods with similar characteristics.^24^ We were unable to ensure whether and how frequently participants were using the stores in their neighborhood. We were also unable to identify those in our cohort who also received care outside the VA and may have been diagnosed with diabetes.

## Conclusions

In this study, neighborhood food environment was associated with type 2 diabetes risk among US veterans in multiple community types, suggesting potential avenues for action to address the burden of type 2 diabetes. Tailored interventions targeting availability of supermarkets may be more appropriate in suburban and rural communities than urban communities, whereas restrictions on fast-food restaurants could possibly help in all community types. These actions, combined with increasing awareness of the risk of type 2 diabetes and the importance of healthy diet intake, might be associated with a decrease in the burden of type 2 diabetes among adults in the US.

## Notes

Supplement. **eAppendix.** *ICD* Codes Used to Identify Type 2 Diabetes **eFigure 1.** Cohort Flow Diagram of Diabetes-Free Cohort of US Veterans, 2008-2016 **eFigure 2.** Piecewise Exponential Models Testing the Effect of Neighborhood Food Environment Density (Count per Square Kilometer) on the Risk of Developing Type 2 Diabetes Among United States Veterans
