# Supplementary material for: Longitudinal Analysis of Neighborhood Food Environment and Diabetes Risk in the Veterans Administration Diabetes Risk Cohort
Source: JAMA Netw Open. 2021 Oct 29;4(10):e2130789. doi: 10.1001/jamanetworkopen.2021.30789 (PMC8556617; doi:10.1001/jamanetworkopen.2021.30789)
Supplement: Supplement. — eAppendix. ICD Codes Used to Identify Type 2 Diabetes eFigure 1. Cohort Flow Diagram of Diabetes-Free Cohort of US Veterans, 2008-2016 eFigure 2. Piecewise Exponential Models Testing the Effect of Neighborhood Food Environment Density (Count per Square Kilometer) on the Risk of Developing Type 2 Diabetes Among United States Veterans [file jamanetwopen-e2130789-s001.pdf]

Supplemental Online Content

Kanchi R, Lopez P, Rummo PE, et al. Longitudinal analysis of neighborhood food environment and diabetes risk in the Veterans Administration Diabetes Risk cohort. *JAMA Netw Open*. 2021;4(10):e2130789. doi:10.1001/jamanetworkopen.2021.30789

eAppendix. *ICD* Codes Used to Identify Type 2 Diabetes

eFigure 1. Cohort Flow Diagram of Diabetes-Free Cohort of US Veterans, 2008-2016

eFigure 2. Piecewise Exponential Models Testing the Effect of Neighborhood Food Environment Density (Count per Square Kilometer) on the Risk of Developing Type 2 Diabetes Among United States Veterans

This supplemental material has been provided by the authors to give readers additional information about their work.

© 2021 Kanchi R et al. *JAMA Network Open.*

eAppendix: *ICD* Codes Used to Identify Type 2 Diabetes:

ICD-9 codes: 250, 250.0, 250.00, 250.02, 250.1, 250.10, 250.12, 250.2, 250.20, 250.22, 250.3, 250.30, 250.32, 250.4, 250.40, 250.42, 250.5, 250.50, 250.52, 250.6, 250.60, 250.62, 250.7, 250.70, 250.72, 250.8, 250.80, 250.82, 250.9, 250.90, 250.92

ICD-10 codes: E11.00, E11.01, E11.21, E11.22, E11.29, E11.311, E11.319, E11.321, E11.3211, E11.3212, E11.3213, E11.3219, E11.329, E11.3291, E11.3292, E11.3293, E11.3299, E11.331, E11.3311, E11.3312, E11.3313, E11.3319, E11.339, E11.3391, E11.3392, E11.3393, E11.3399, E11.341, E11.3411, E11.3412, E11.3413, E11.3419, E11.349, E11.3491, E11.3492, E11.3493, E11.3499, E11.351, E11.3511, E11.3512, E11.3513, E11.3519, E11.352, E11.3521, E11.3522, E11.3523, E11.3529, E11.353, E11.3531, E11.3532, E11.3533, E11.3539, E11.354, E11.3541, E11.3542, E11.3543, E11.3549, E11.355, E11.3551, E11.3552, E11.3553, E11.3559, E11.359, E11.3591, E11.3592, E11.3593, E11.3599, E11.36, E11.37, E11.37X1, E11.37X2, E11.37X3, E11.37X9, E11.39, E11.40, E11.41, E11.42, E11.43, E11.44, E11.49, E11.51, E11.52, E11.59, E11.610, E11.618, E11.620, E11.621, E11.622, E11.628, E11.630, E11.638, E11.641, E11.649, E11.65, E11.69, E11.8, E11.9

© 2021 Kanchi R et al. *JAMA Network Open.*

eFigure 1. Cohort Flow Diagram of Diabetes-Free Cohort of US Veterans, 2008–2016

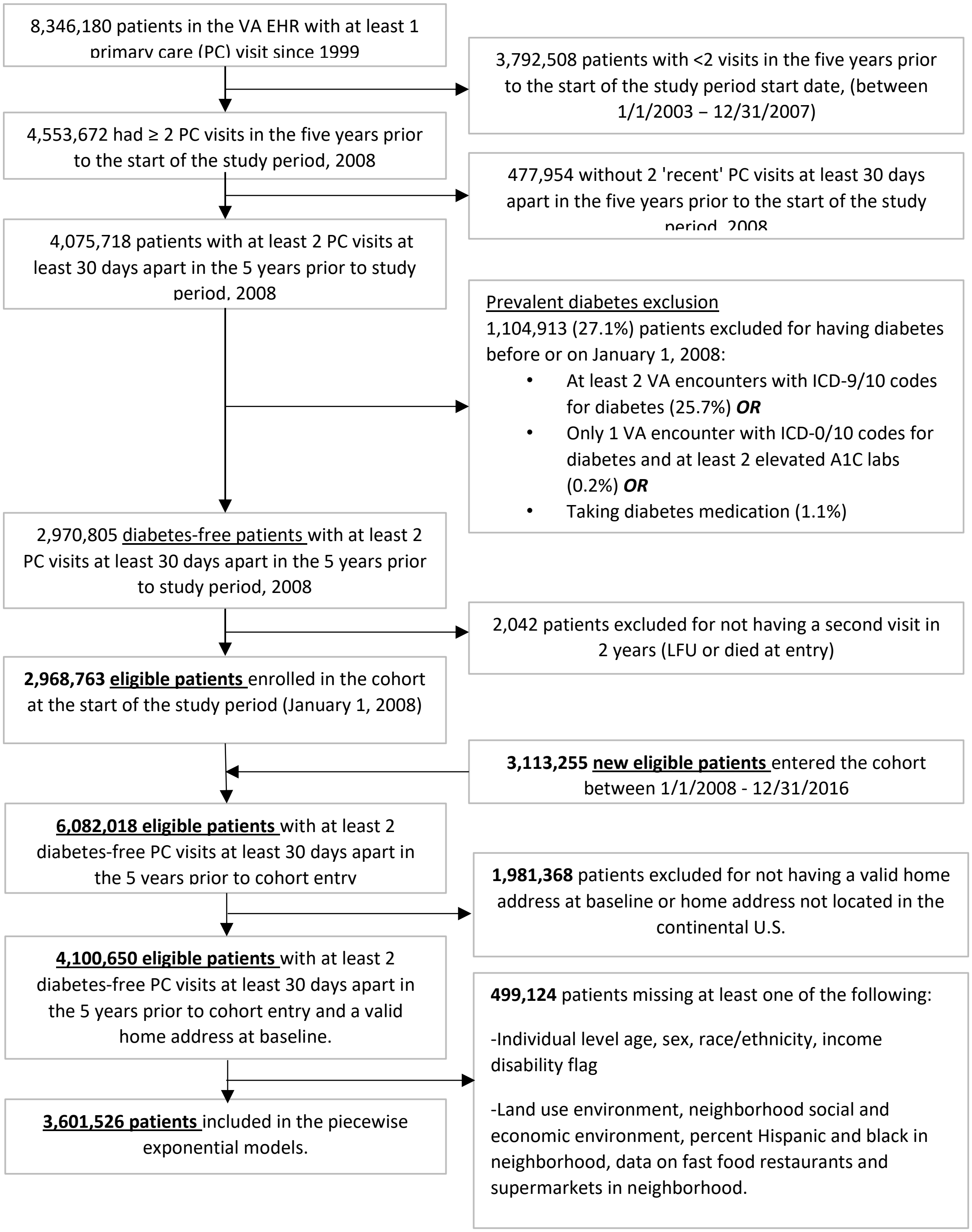

© 2021 Kanchi R et al. *JAMA Network Open.*

eFigure 2: Piecewise Exponential Models Testing the Effect of Neighborhood Food Environment Density (Count per Square Kilometer) on the Risk of Developing Type 2 Diabetes Among United States Veterans

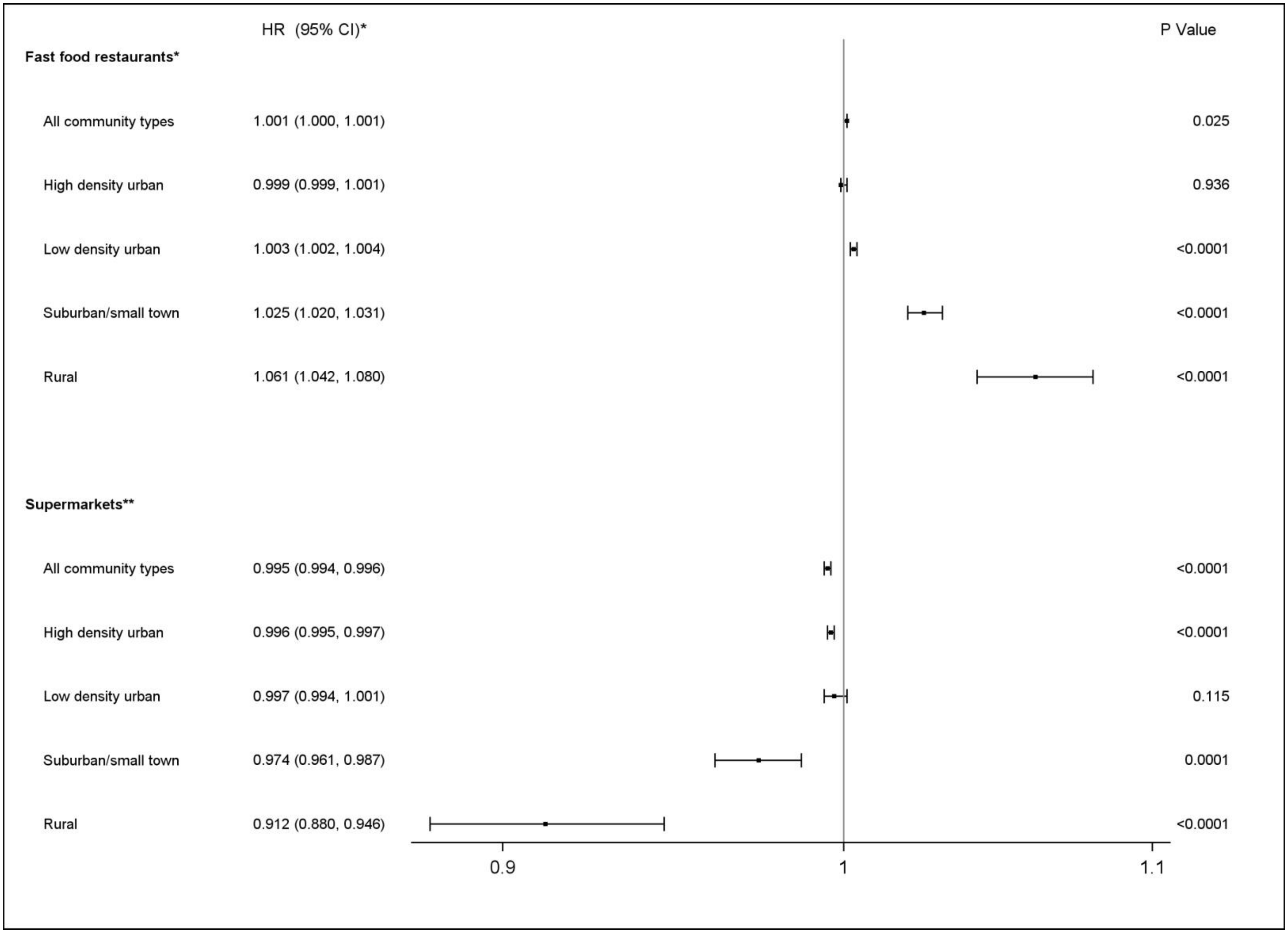

* Model adjusted for age, sex, race/ethnicity, income/disability flag, land use environment, neighborhood social and economic environment, percent Hispanic and black in neighborhood, and 5-year average supermarket density in network buffers.

** Model adjusted for age, sex, race/ethnicity, income/disability flag, land use environment, neighborhood social and economic environment, and percent Hispanic and black in neighborhood.

Only individuals with available data on all the variables in the models are included (n=3,601,526).

© 2021 Kanchi R et al. *JAMA Network Open.*
